# Non-Linear Characterisation of Cerebral Pressure-Flow Dynamics in Humans

**DOI:** 10.1371/journal.pone.0139470

**Published:** 2015-09-30

**Authors:** Saqib Saleem, Paul D. Teal, W. Bastiaan Kleijn, Terrence O’Donnell, Trevor Witter, Yu-Chieh Tzeng

**Affiliations:** 1 School of Engineering and Computer Science, Victoria University of Wellington (VUW), Wellington, New Zealand; 2 Cardiovascular Systems Laboratory, Centre for Translational Physiology, University of Otago (UO), Wellington, New Zealand; Medical University of Graz, AUSTRIA

## Abstract

Cerebral metabolism is critically dependent on the regulation of cerebral blood flow (CBF), so it would be expected that vascular mechanisms that play a critical role in CBF regulation would be tightly conserved across individuals. However, the relationships between blood pressure (BP) and cerebral blood velocity fluctuations exhibit inter-individual variations consistent with heterogeneity in the integrity of CBF regulating systems. Here we sought to determine the nature and consistency of dynamic cerebral autoregulation (dCA) during the application of oscillatory lower body negative pressure (OLBNP). In 18 volunteers we recorded BP and middle cerebral artery blood flow velocity (MCAv) and examined the relationships between BP and MCAv fluctuations during 0.03, 0.05 and 0.07Hz OLBNP. dCA was characterised using project pursuit regression (PPR) and locally weighted scatterplot smoother (LOWESS) plots. Additionally, we proposed a piecewise regression method to statistically determine the presence of a dCA curve, which was defined as the presence of a restricted autoregulatory plateau shouldered by pressure-passive regions. Results show that LOWESS has similar explanatory power to that of PPR. However, we observed heterogeneous patterns of dynamic BP-MCAv relations with few individuals demonstrating clear evidence of a dCA central plateau. Thus, although BP explains a significant proportion of variance, dCA does not manifest as any single characteristic BP-MCAv function.

## Introduction

**Fig 1:**
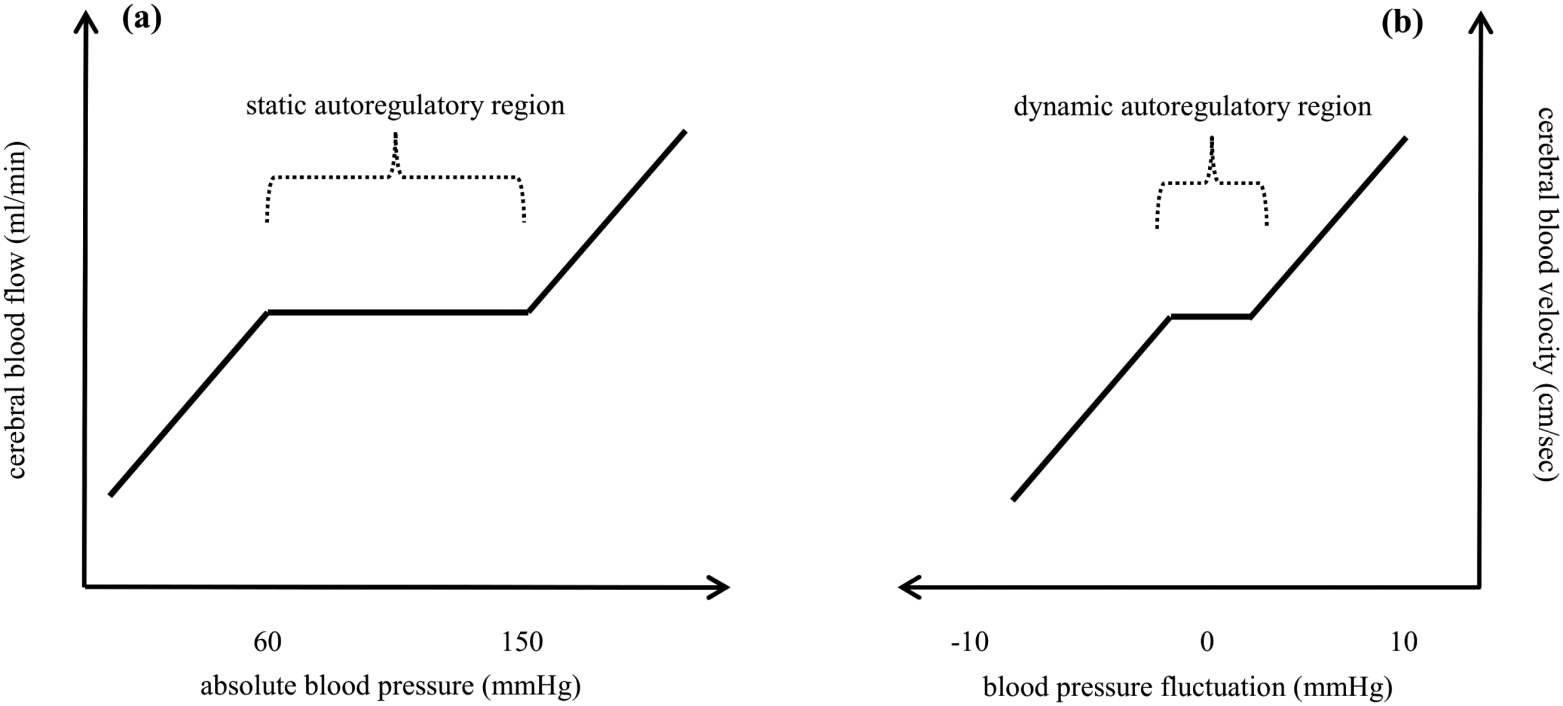
Stylised representation of cerebral autoregulatory behaviour. ***(a)*** Lassen’s classical autoregulatory curve of static cerebral autoregulation [7], ***(b)*** nonlinear curve for dynamic cerebral autoregulation (dCA) [4,15].

The human brain is a highly metabolically active organ that comprises only 1–2% of total body weight, but accounts for 20% of resting total body O_2_ consumption [1]. Because of this high demand for energy, stringent regulation of cerebral blood flow (CBF) is paramount for normal brain function and several mechanisms have been identified as key regulators of CBF homeostasis [2–4]. One of these mechanisms is cerebral autoregulation (CA), which refers to the active dilation and constriction of the cerebral resistance blood vessels in response to changes in cerebral perfusion pressure [3, 5, 6]. Using system-level terminology, CA is often thought to be composed of both dynamic and steady-state response components. The ‘static’ component refers to CBF regulations against gradual BP changes over minutes to hours and is encapsulated by Lassen’s classic autoregulatory curve [7], which has a characteristically wide central CBF plateau for BP levels ranging between 60 and 150 mmHg (Fig 1a). In contrast, the ‘dynamic’ component (dCA) typically refers to rapid cerebrovascular responses to BP transients occurring over seconds [8, 9].

Cerebral metabolism is critically reliant on a stable blood supply, so it is generally assumed that vital mechanisms such as dCA are tightly conserved across healthy individuals within a population [2, 3]. However, in practice the dynamic relationships between BP and cerebral blood velocity fluctuations exhibit marked inter-subject variations [10], suggesting that the functional integrity of CBF regulating systems such as dCA may vary significant between individuals. Unfortunately, gaining insight into the nature of such variations is challenging since dCA is a nonlinear process and the use of linear methods such as transfer function analysis [6, 11], autoregressive models [12, 13], and linear differential equations [14] may not accurately capture the true nature of dCA.

Recent studies characterised the oscillatory relationships between BP and middle cerebral blood flow velocity (MCAv) using nonlinear projection pursuit regression (PPR) [4, 15]. It was reported that a characteristic signature of dCA at frequencies between 0.03–0.07 Hz is the presence of a restricted (~5–10 mmHg range) autoregulatory plateau shouldered by pressure-passive regions (Fig 1b; herein referred to simply as the dCA curve). However, whilst such a nonlinear curve might directly reflect dCA, we were motivated to evaluate the validity of this construct for at least two reasons. First, the concept suggests that dCA effectively responds against only minor deviations (5 mmHg) in the very low frequency (VLF) component of BP. This is a surprising inference since BP deviations within 5 mmHg do not present a major threat to cerebral metabolism and microscopy studies have shown that pial precapillary arterioles typically do not respond to BP changes unless deviations exceed >10–15 mmHg relative to baseline values (i.e., when there is a clear stimulus and threat to cerebral perfusion) [16]. Second, the concept implies that BP deviations exceeding narrow dCA margins are associated with parallel changes in CBF. This is inconsistent with experimental data suggesting that precapillary arterioles can undergo active dilation and constriction across a wide BP range [16]. Thus in our view, dynamic cerebral autoregulation in healthy subjects may not necessarily conform to the dCA curve reported in recent studies [4, 15].

Here we report findings from an experimental series that sought to test the presence and consistency of the dCA curve across different subjects during the application of oscillatory lower body negative pressure (OLBNP). To date the dCA curve has only been observed using PPR [4, 15], which is only one of the several methodologies that can theoretically accommodate the characterisation of nonlinear relationships. The validity of the dCA curve would be greatly strengthened if it could be replicated using complementary methods. Therefore, we deployed both PPR as well as locally weighted scatterplot smoothing (LOWESS) [17, 18] analyses on BP and MCAv time series collected from healthy individuals. Additionally, we also developed and applied a statistical approach based on piecewise linear regression to test the hypothesis that the BP-MCAv fluctuations contain a central dCA plateau.

## Materials and Methods

### Subjects

Eighteen healthy, normotensive and non-smoking subjects (9 women; mean age 23 0.67 years) were recruited for this study. This study conformed to the standards set by the Declaration of Helsinki and was approved by the New Zealand Central Regional Ethics Committee. All subjects were advised to abstain from caffeine-containing beverages and heavy exercise for at least 12 hours before the study and to have a light breakfast at least 2 hours before the study. None of the subjects was on regular medication and none had a known history of respiratory, cardiovascular or endocrine diseases. All subjects gave written informed consent prior to participation.

### Measurements

A data-acquisition unit (Powerlab/16 SP ML 795, AD Instruments) was used to record electrocardiogram (ECG), non-invasive beat-to-beat blood pressure via the finger photoplethysmography (Finometer, MIDI, MLE1054-V, Finapres Medical Systems, Amsterdam, Netherlands), right middle cerebral artery blood flow velocity (MCAv; 2MHz pulsed Doppler Ultrasound, ST3 Transcranial Doppler (TCD), Spencer Technologies) and end-tidal CO_2_ sampled from a nasal line (Gas analyser model ML206, AD Instruments, Colorado Springs, CO, USA) at 1 kHz per channel. The data was stored on a computer for off-line analysis. For TCD assessment, a headband strap (Marc 600, Spencer Technologies) was used to maintain optimal insonation angle throughout each testing session.

### Experimental Protocols

This study was conducted in the supine position in a quiet, temperature- and humidity-controlled laboratory (22–23°) between 9 am-12 noon. Following approximately 10 minutes of stabilization, we recorded 10 min of resting data before commencing a comprehensive BP manipulation protocol involving OLBNP, bilateral thigh cuff deflation and sit-to-stand manoeuvres in the upright position. Findings for the thigh cuff deflation and sit-to-stand experiments were designed to test distinct *a priori* defined hypotheses that have already been reported elsewhere [19]. For the OLBNP protocol, subjects were studied with their lower body sealed in a tank connected to a vacuum source. During testing, the vacuum source was set to periodically oscillate the chamber pressure between 0 and approximately -45 mmHg at 0.03, 0.05 and 0.07Hz in randomized order. In two subjects a slightly greater negative pressure of -60 mmHg was applied to generate comparable and consistent fluctuations in BP. These frequencies were chosen because they lie within the range where dCA is commonly thought to be operant [20, 21]. At each OLBNP frequency, 12 cycles were completed before transition to the next frequency, except for two subjects where 15 cycles were completed. For subsequent analysis 12 cycles were analysed across each OLBNP frequency for all subjects. To ensure accurate measurements, finger blood pressure recordings were always recalibrated before applying OLBNP and were verified against the manually measured brachial artery blood pressure.

### Signal pre-processing

Data were analysed using custom written software in MATLAB (version R2014b; Mathworks) and R-Language (version 3.0.1; R foundation for statistical computing, Vienna, Austria). In keeping with previous studies [4, 15], the 1-kHz recorded continuous BP and MCAv (an index of CBF) waveforms were averaged over each cardiac interval. These reduced time-series were cubic-spline interpolated and then down-sampled to 5-Hz before linear detrending. A low pass anti-aliasing filter was also used to avoid aliasing while down-sampling the interpolated time-series. Grubb’s test was employed to identify and remove occasional measurement artefacts [22].

To account for the effects of different sampling rates and filters on the characteristic relationship between BP and MCAv signals, we applied several alternative data processing methods. First, in keeping with previous studies [4, 15], we analysed 5-Hz time-series data that were bandpass filtered with a bandwidth of 0.01 Hz centred at OLBNP frequency to remove the effects of random fluctuations. Second, given that 5-Hz time series tend to exhibit high serial correlation, we repeated our analyses on BP and MCAv time series that were first decimated to 0.23-Hz to remove the potential confounding influence of serial correlation before bandpass filtering. This lower sampling frequency corresponded to a small fraction of the bandwidth of the spontaneous BP and MCAv fluctuations, and was well below the cardiac (0.6–1 Hz) and the breathing frequencies (0.3–0.37 Hz). Examination of the autocorrelation functions suggest that time series resampled at this rate were largely decorrelated having only 0.13 0.03 auto-correlation on average. Finally, since nonlinear BP-MCAv relations may generate harmonics associated with the induced OLBNP frequency, we also assessed the effects of applying a comb filter to the 0.23-Hz resampled data to determine the effects of these harmonics on resultant BP-MCAv relations.

Transient artefacts at the beginning of all time series associated with data filtering were removed by truncating the first *n* samples (where *n* is the filter length). In keeping with previous studies [4, 15], all analyses were conducted with time series that were synchronised to each other without any inherent delays. However, to account for the effects of potential delays between BP and MCAv dynamics, we identified the maximum BP-MCAv cross-correlation and also analysed signals that were realigned according to this delay. Results for these sensitivity analyses are presented as supplementary information (see S3 File).

### Projection pursuit regression (PPR)

PPR is essentially a non-parametric regression approach that models the regression curve as a sum of general smooth functions (termed ‘ridge functions’) of linear combinations of the predictor variables [23]. In keeping with previous studies [4, 15], PPR models were specified to include only one ridge function, which can be visually interpreted as the relation between BP and MCAv that best fits the data. The detailed description and mathematical formulation of PPR can be found elsewhere [23].

### LOWESS

In this study, we employed LOWESS as a complementary nonlinear analytic method to capture the intrinsic nonlinearities of the relationship between fluctuations in BP and MCAv without imposing any *a-priori* assumptions about the input-output relationship [17]. A full description of LOWESS can be found elsewhere [17] but briefly LOWESS is a local polynomial based regression technique that characterizes the relationships between paired variables by fitting a smooth curve to the scatter diagram of data points. The algorithm begins by first determining the local neighbourhood of a given data point before applying a weighting function to its neighbourhood and then fitting a local linear polynomial to the weighted points within that neighbourhood. This procedure is repeated for all data points of the input series to derive estimated points for the output, which are then joined using linear local polynomials to obtain a smooth fitted curve.

### Piecewise regression

In this study we adopted two variants of piecewise linear regression for the statistical detection of central MCAv plateaus that may be indicative of a dCA curve. In keeping with previous studies [4, 15], the first approach uses the estimated models of PPR and LOWESS, and fits three hinged straight lines using Bruno-Luong’s Free-knot spline approximation technique. The mathematical formulation and implementation details of Bruno-Luong’s Free-knot spline approximation can be found elsewhere [24] but briefly this parameterization statistically determines the points where the pressure-flow relationships changes and approximates separate regions by linear functions.

The second approach fits three unhinged straight lines to the BP-MCAv data points rather than to the estimated models of PPR or LOWESS (see S1 File for full derivation). Here the same data segments (of hinged regression) are used and a least square line is fitted to each segment. The least square line between BP and MCAv suggests a model of the form [25]

$$V\left(n\right)=\beta_{o}+\beta_{1}P\left(n\right)+\epsilon \left(n\right)$$ (1)

where *V*(*n*) is MCAv, *P*(*n*) is BP, *ϵ*(*n*) is the error in the *n*-th observation and, *β*_*0*_ and *β*_*1*_ are the intercept and the gradient of the least square line, respectively. We can calculate the mean of *β*_*1*_ and its uncertainty under the following assumptions concerning the errors in the least-square model [25]: (1) the errors are random and independent, (2) the errors have the mean zero, (3) the errors have the same variance, and (4) the errors are normally distributed. Under these assumptions, the estimated quantities ${\hat{\beta }}_{o}$ and ${\hat{\beta }}_{1}$ become normally distributed random variables. The use of 0.23-Hz resampled data ensures these assumptions are fulfilled. As we are interested only in the gradient ${\hat{\beta }}_{1}$, its mean and standard deviation can be obtained by equation (A1) and equation (A2) given in S1 File. From the means and the standard deviations of the normally distributed gradients of the least-square lines, we can plot their probability distributions, which describe the BP-MCAv dynamics in terms of probability distributions. Where BP-MCAv relationships resemble the dCA curve, the gradient of the middle (i.e., autoregulatory) segment should be lower than that of the right (higher BP) and the left (lower BP) segments. Accordingly, the probability distribution of the middle segment will show relative shift towards low blood pressure values (i.e., leftward displacements) as compared to the probability distributions of the left- and the right segments. The test statistic for unhinged regression has a Student’s *t* distribution with *N*-3 degrees of freedom, where *N* is the number of data points in each segment of time series (see S1 File for details).

Although serial correlation was largely eliminated by down-sampling the data to 0.23-Hz, it remains possible that residual serial correlation could affect the estimated probability distributions of unhinged regression lines. We accounted for such effects by considering the serial correlation factor defined in equation (A4).

Additionally, we developed and applied a statistical approach to determine the significance of the estimated gradients of piecewise regressions by examining the hypothesis defined in equation (A6). The P-values corresponding to the test-statistics, defined in equation (A7) for hinged regression and in equation (A8) for unhinged regression, were determined to validate the statistical significance of the null and alternate hypothesis. Here, the P-values corresponding to the null hypothesis for hinged regression are determined using an empirical approach by generating multiple observations of the normal data with the same statistical features (i.e., mean, standard deviation and correlation) as that of the original BP-MCAv data.

### Power spectral analysis

Spectral analysis of BP and MCAv time series was performed based on Welch’s overlapped segment averaging estimator. First, each bandpass filtered time series was divided into 5 segments (each segment consisting of 2–3 cycles of OLBNP) with 50% overlap between the consecutive segments. A Hann window was then applied to each data segment before fast Fourier transform analysis. The modified periodograms were averaged to obtain power spectral density (PSD) estimates. The cross spectrum between BP and MCAv was divided by the auto spectrum of BP from which the coherence, gain and phase indexes were derived.

### Statistics

All values are presented as means±SE unless otherwise stated. PSD values were log transformed, coherence values were r-to-z transformed and phase values were arcsine transformed to get asymptotical distributions. R^2^ values were transformed to normally distributed values using Box-Cox transformation. The normal distribution of the data was established by Shapiro-Wilk test. For ease of interpretation and analysis, all values and confidence intervals are presented here in standard units. All comparisons were made using either one- or two-way repeated measures ANOVA tests. For two-way ANOVA, the experimental condition (i.e., OLBNP frequency) and regression approach were adopted as independent factors while for one-way ANOVA the experimental condition (i.e., OLBNP frequency) was considered as the independent factor. When a significant interaction between OLBNP frequency and regression approach was observed, two-way ANOVA was followed by post hoc Tukey’s honestly significant difference test and one-way ANOVA was followed by a post-hoc *paired-t* test to determine at which OLBNP frequency significant difference was present. The confidence interval for Grubb’s test was 0.05. The variance-explained is defined as R^2^ = 1—SS_E_/ SS_T_, where SS_E_ is sum of squared errors and SS_T_ is sum of squared true values. Cross-validation of regression techniques was performed using a leave-one-out approach (i.e., one data point is used for evolution of a model fitted to the remaining data points within each subject). Unless otherwise stated statistical significance was set *a priori* at P < 0.05.

## Results

**Fig 2:**
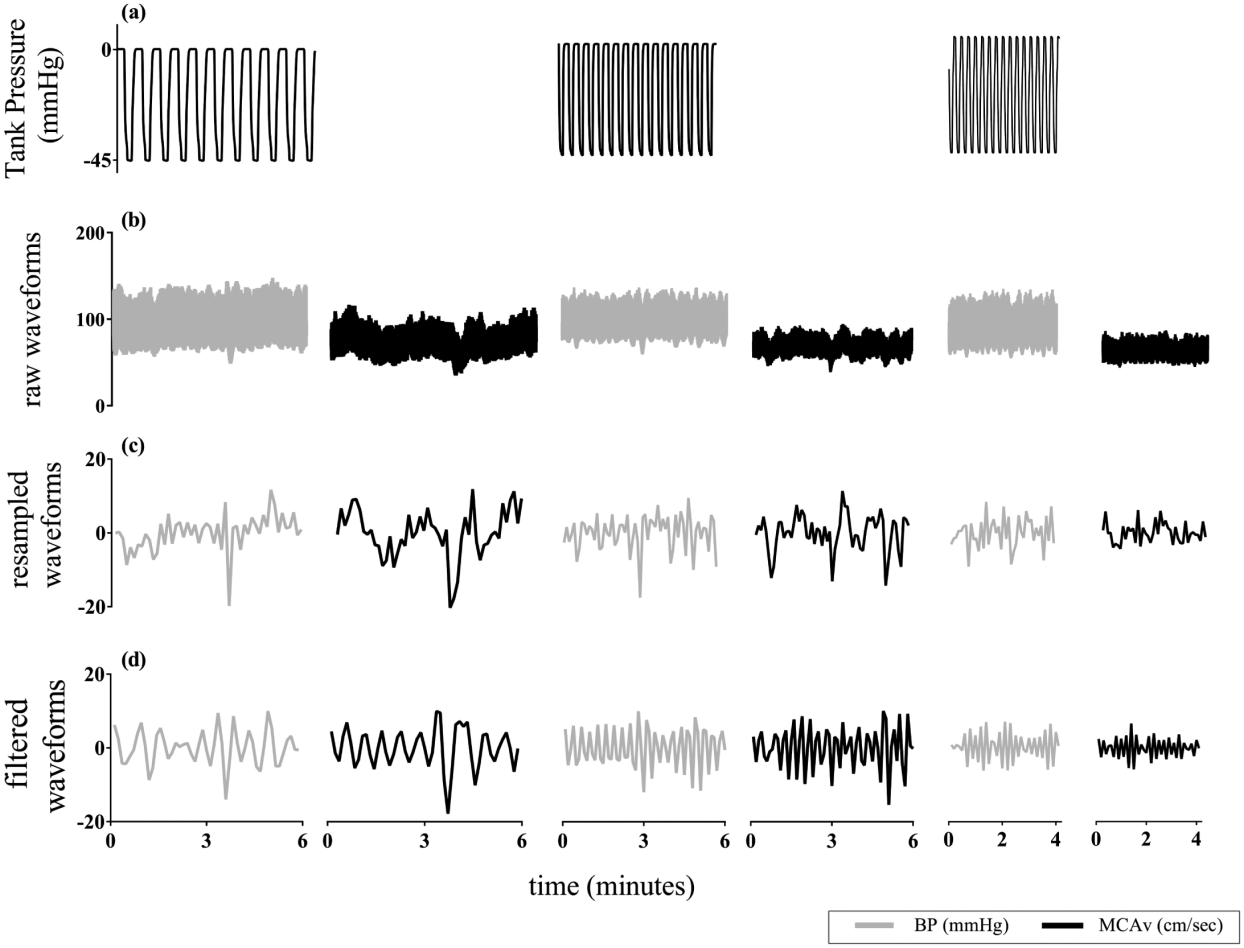
Representative example of the study protocol showing tank pressure, and raw, 0.23-Hz resampled and band-pass filtered (0.01 Hz bandwidth) BP and MCAv time series (subject 1). ***(a)*** tank pressure (mmHg); leftmost is 0.03Hz, middle is 0.05Hz and rightmost is 0.07Hz OLBNP, ***(b)*** raw BP and MCAv time series, sampled at 1-KHz, ***(c)*** zero-mean 0.23-Hz resampled BP and MCAv time series, ***(d)*** band-pass filtered (0.01 Hz bandwidth) BP and MCAv time series. OLBNP, oscillatory lower body negative pressure; BP, blood pressure; MCAv, middle cerebral artery blood flow velocity.

Cardiovascular, respiratory and cerebrovascular parameters for OLBNP protocols are presented in Table 1. We observed that all haemodynamic variables were similar across the three OLBNP conditions (P > 0.28 for all comparisons; one-way repeated measures ANOVA). An illustrative example of raw BP and MCAv, and corresponding 0.23-Hz resampled and bandpass filtered (using 0.01 Hz bandwidth filter) time series for three OLBNP frequencies along with the input OLBNP signal (i.e., tank pressure) is shown in Fig 2. The following sections present 5-Hz and 0.23-Hz resampled data that was bandpass filtered (0.01 Hz bandwidth). Summary results of delay-shifted data and subject to comb filtering are presented as supporting information (see S3 File).

**Table 1:** Haemodynamic Variables.

	OLBNP frequency (Hz)		
Variable	0.03	0.05	0.07
R-R Interval (sec)	1 ± 0.02	1 ± 0.03	1 ± 0.03
Breathing rate (breath.min^-1^)	13 ± 0.97	13 ± 0.63	14 ± 0.92
End-tidal CO_2_ (mmHg)	38 ± 0.55	39 ± 0.82	39 ± 0.67
Systolic BP (mmHg)	128 ± 3	130 ± 1	134 ± 2
Diastolic BP (mmHg)	58 ± 2	58 ± 2	57 ± 1
MAP (mmHg)	82 ± 2	83 ± 1	82 ± 1
MCAv_mean_ (cm.s^-1^)	64 ± 4	64 ± 4	65 ± 4

Values are mean ± SE for OLBNP data. R-R Interval, beat-to-beat interval; MAP, mean arterial pressure; MCAv_mean_, mean middle cerebral artery blood flow velocity; OLBNP, oscillatory lower body negative pressure. P > 0.28 for all comparisons; one-way repeated measures ANOVA.

### Power spectral analysis

Table 2 shows power spectral densities and Table 3 shows the cross-spectral coherence, gain and phase associated with each OLBNP frequency. Overall coherence during OLBNP was found to be below than 0.4 in 8 subjects at 0.03Hz OLBNP, in 4 subjects at 0.05Hz, and in 2 subjects at 0.07Hz. We found that the coherence between BP and MCAv fluctuations increased with increase in OLBNP frequency (P < 0.05; one-way repeated measures ANOVA). Gain values showed a tendency to increase with higher OLBNP frequency (P < 0.05; one-way repeated measures ANOVA). In contrast, phase decreased with higher OLBNP frequencies (P < 0.05; one-way repeated measures ANOVA).

**Table 2:** Power spectral density of BP and MCAv during OLBNP for 0.23-Hz resampled data.

	OLBNP frequency (Hz)		
Variable	0.03	0.05	0.07
BP spectral density (mmHg^2^ Hz^-1^)	3.39 ± 0.96	3.69 ± 1.06	3.28 ± 0.94
MCAv spectral density (cm^2^ s^-2^ Hz^-1^)	2.57 ± 0.4	1.43 ± 0.45	2.57 ± 0.43

Values are mean ± SE for OLBNP data. BP, blood pressure; MCAv, middle cerebral artery blood flow velocity; OLBNP, oscillatory lower body negative pressure. P > 0.15 for all comparisons; one-way repeated measures ANOVA.

**Table 3:** Cross-spectral coherence, gain and phase for 0.23-Hz resampled BP and MCAv during OLBNP.

	OLBNP frequency (Hz)		
Variable	0.03	0.05	0.07
Coherence (AU)	0.42 ± 0.06	0.52 ± 0.059*	0.64 ± 0.05* †
Gain (cm/s/mmHg)	0.29 ± 0.04	0.43 ± 0.04*	0.52 ± 0.03* †
Phase (degrees)	46 ± 14	28 ± 14*	20 ± 7* †

Values are mean ± SE for OLBNP data. AU, arbitrary units; BP, blood pressure; MCAv, middle cerebral artery blood flow velocity; OLBNP, oscillatory lower body negative pressure.

* P < 0.05 vs. 0.03Hz

^†^ P < 0.05 vs. 0.05Hz for significant differences; one-way repeated measures ANOVA.

### Analysis of 5-Hz resampled data

**Fig 3:**
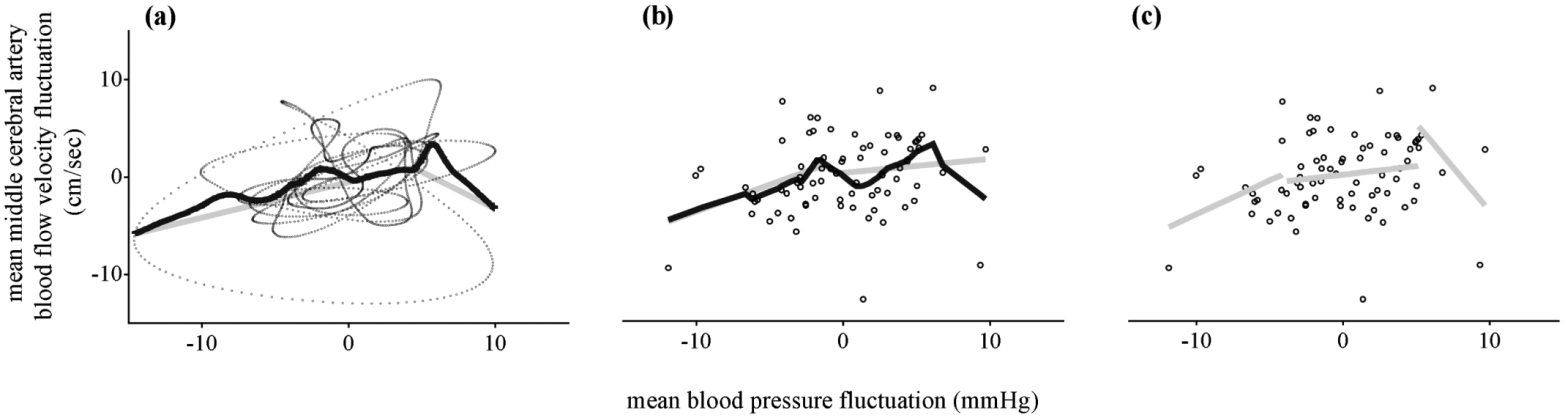
Example of LOWESS fitted curve and two piecewise regression approaches for 0.03Hz OLBNP (subject 1 in Fig 4) superimposed on individual BP-MCAv data points. Dots represent BP and MCAv data points. ***(a)*** LOWESS fitted curve to 5-Hz data points along with hinged regression lines, ***(b)*** LOWESS fitted curve to 0.23-Hz data points along with hinged regression lines, **(c)** unhinged piecewise regression lines to 0.23-Hz data points. LOWESS, locally weighted scatterplot smoother; BP, blood pressure; MCAv, middle cerebral artery blood flow velocity; OLBNP, oscillatory lower body negative pressure.

**Fig 4:**
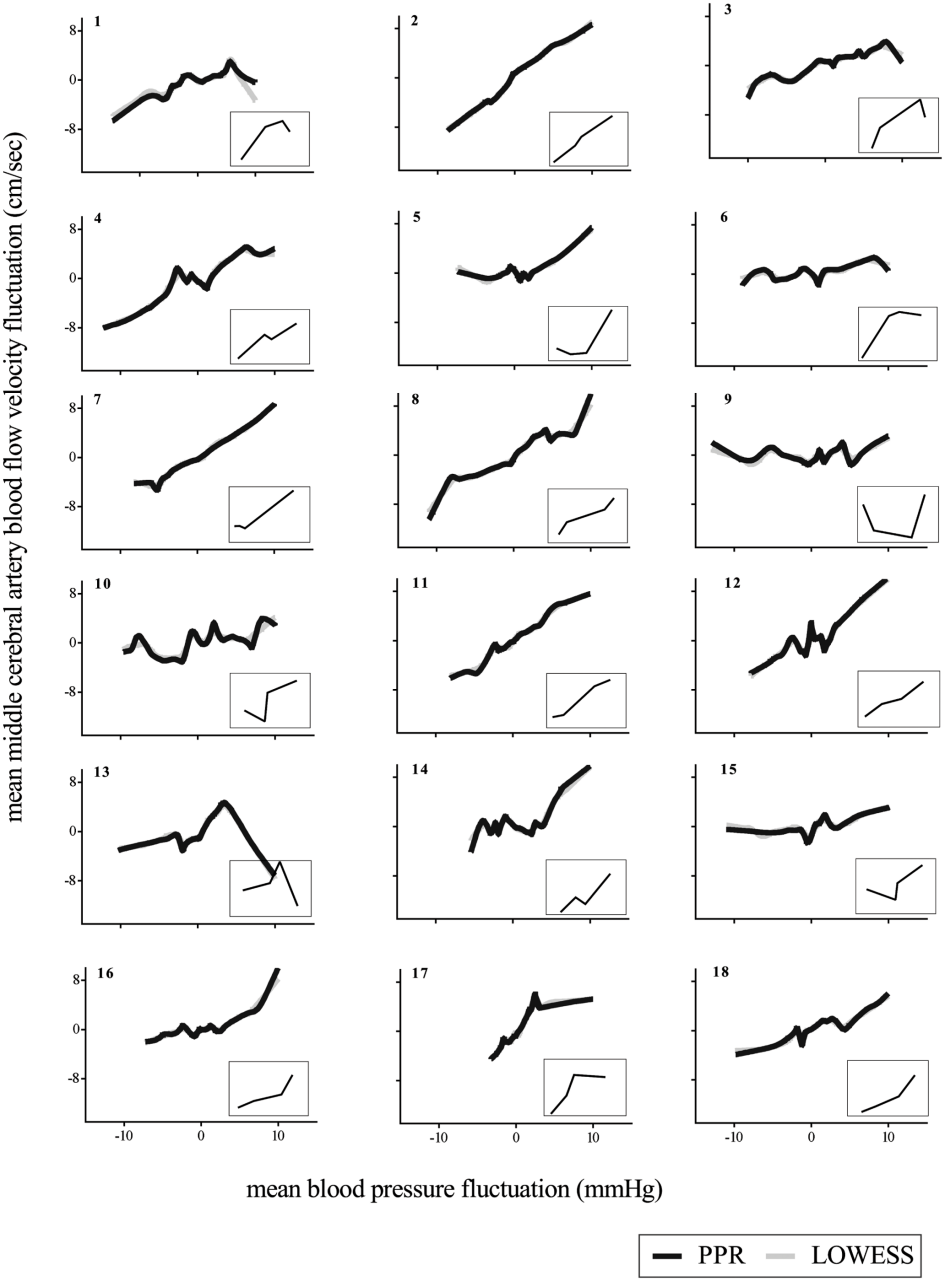
LOWESS and PPR estimated models of 5-Hz resampled data for 0.03Hz OLBNP. The corresponding hinged lines to PPR curves using Bruno-Luong’s Free-knot spline approximation are shown in inset of each subplot. Along axis, mean values of BP and MCAv fluctuations are given. In each subplot the top-left digit(s), ***italic-bold***, refers to the subject’s identifier. BP, blood pressure; MCAv, middle cerebral artery blood flow velocity; OLBNP, oscillatory lower body negative pressure; PPR, projection pursuit regression; LOWESS, locally weighted scatterplot smoother.

Fig 3a shows an example of LOWESS curve fitted to 5-Hz resampled data at 0.03Hz OLBNP. Fig 4 shows the characteristic relationships between BP and MCAv derived using PPR and LOWESS at 0.03Hz OLBNP. Visual inspection of the derived curves indicate that both techniques yield qualitatively similar curves with comparable explanatory power in terms of proportion of MCAv variance explained by the models (46.93±4.6% by PPR vs. 50±4.2% by LOWESS; see S1 Fig and S2 File for more details).

Insets of Fig 4 show the corresponding hinged piecewise regression lines fitted to PPR curves. In summary, at low BP (i.e., left segment) 14 subjects had positive gradients, 1 subject had negative gradient and 3 subjects had approximately zero gradient (P > 0.05 for linear regression). For the middle segment, 9 subjects had positive gradients, 4 subjects had negative gradients and 5 subjects have approximately zero gradients (P > 0.05 for linear regression). At high BP (i.e., right segment) 16 subjects had positive gradients, 1 subject had negative gradient, and 1 subject had approximately zero gradient (P > 0.05 for linear regression). Thus, autoregulatory regions (i.e., slope approximately zero) were identified in several subjects, but when all three segments are taken into account only 5 individuals exhibited patterns that clearly resembled the dCA curve.

The P-values of the test statistic (defined in equation A7) for hinged piecewise regression were greater than 0.05 for all subjects, except for three subjects for which the middle segment gradient is negative and large (see S1 Table). Similar results were obtained for piecewise regression on LOWESS curves (see S2 Fig). Collectively, these observations show that the hinged lines fitted to PPR and LOWESS curves did not reveal any consistent BP-MCAv relationships that resemble the dCA curve.

### Analysis of 0.23-Hz resampled data

Data series sampled at 5-Hz exhibit high serial correlation, so BP-MCAv relationships were also assessed on 0.23-Hz resampled time series where serial correlation was suppressed. Similar to the results based on 5-Hz data, we found that BP-MCAv relationships characterised using LOWESS were similar to those with PPR with comparable MCAv variance explained (47.5±5.28% by PPR vs. 52±4.5% by LOWESS; see S1 Fig and S2 File for details).

**Fig 5:**
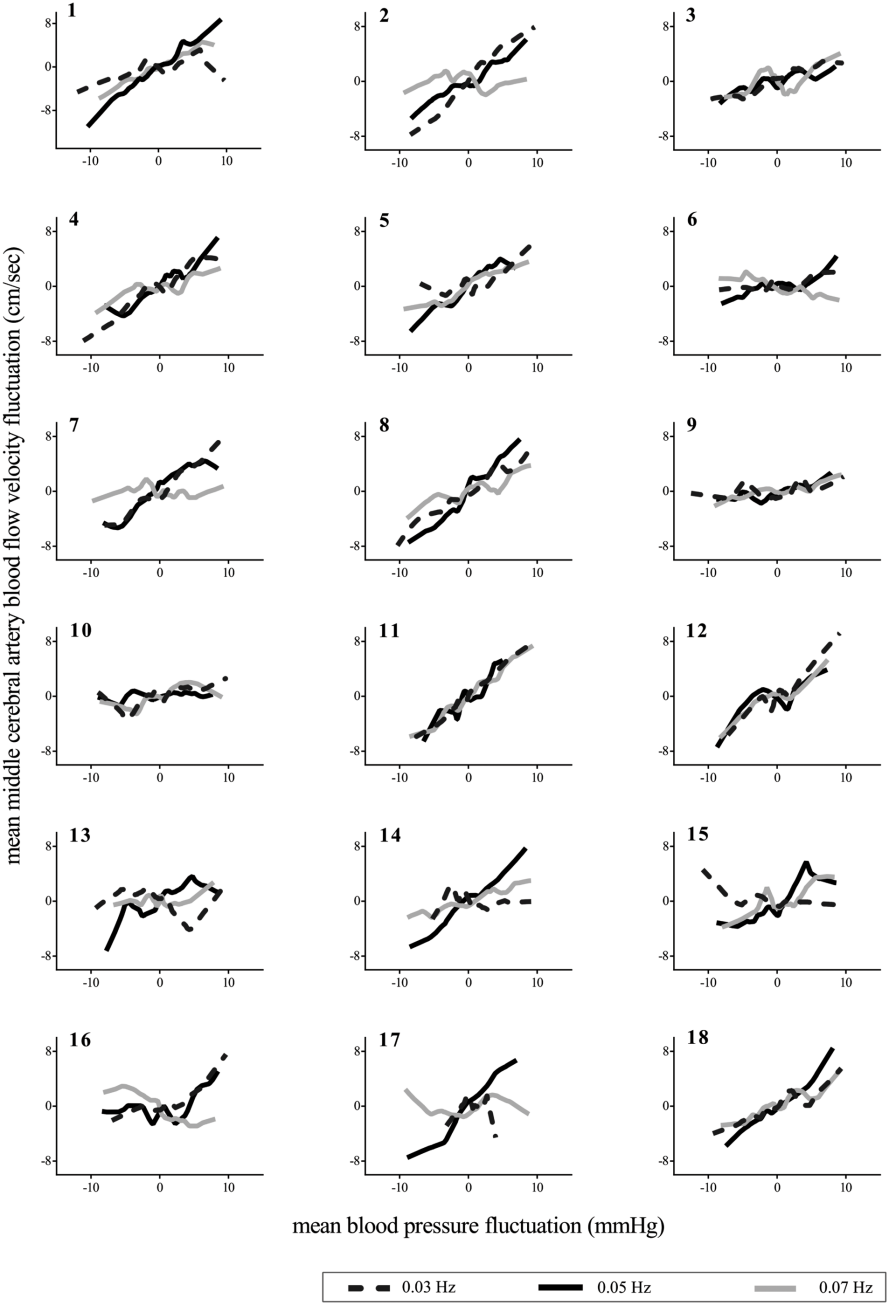
LOWESS estimated models of 0.23-Hz resampled OLBNP data. Along axis, mean values of BP and MCAv fluctuations are given. In each subplot the top-left digit(s), ***italic-bold***, refers to the subject’s identifier. LOWESS, locally weighted scatterplot smoother; OLBNP, oscillatory lower body negative pressure; BP, blood pressure; MCAv, middle cerebral artery blood flow velocity.

Fig 3b shows an illustrative example of LOWESS curve fitted to 0.23-Hz resampled BP and MCAv data at 0.03 Hz OLBNP, and Fig 5 summarises LOWESS curves for all subjects at each of the three OLBNP frequencies. In general we observed marked diversity in the characteristic relationships between BP and MCAv with the vast majority of subjects showing asymmetric BP-MCAv relationships around the mean values. At 0.03Hz OLBNP, BP was positively related to MCAv in the majority of subjects, however in 8 cases a negative relation was found at BP extremes. Qualitative assessment across all subjects showed that only 14 out of 54 cases (i.e., 3 OLBNP frequencies for each subject) showed BP-MCAv relations that clearly resembled a central MCAv plateau (e.g., subject 12). However, in these individuals MCAv did not necessarily become more positively linear at higher OLBNP frequencies. For example, in some individuals the BP-MCAv relationships were (positively) linear at 0.03Hz OLBNP but resembled a flat plateau at 0.05 or 0.07Hz (e.g., subject 2).

To statistically establish the possible presence of a central dCA plateau based on these LOWESS curves (of Fig 5), we fitted hinged piecewise regression lines to each curve. Fig 3b shows a representative example of piecewise hinged regressions lines fitted to LOWESS curves at 0.03Hz OLBNP. Overall we found that the P-values of the test statistic (defined in equation A7) for hinged regression were greater than 0.05 for all subjects consistent with the absence of a central dCA plateau.

**Fig 6:**
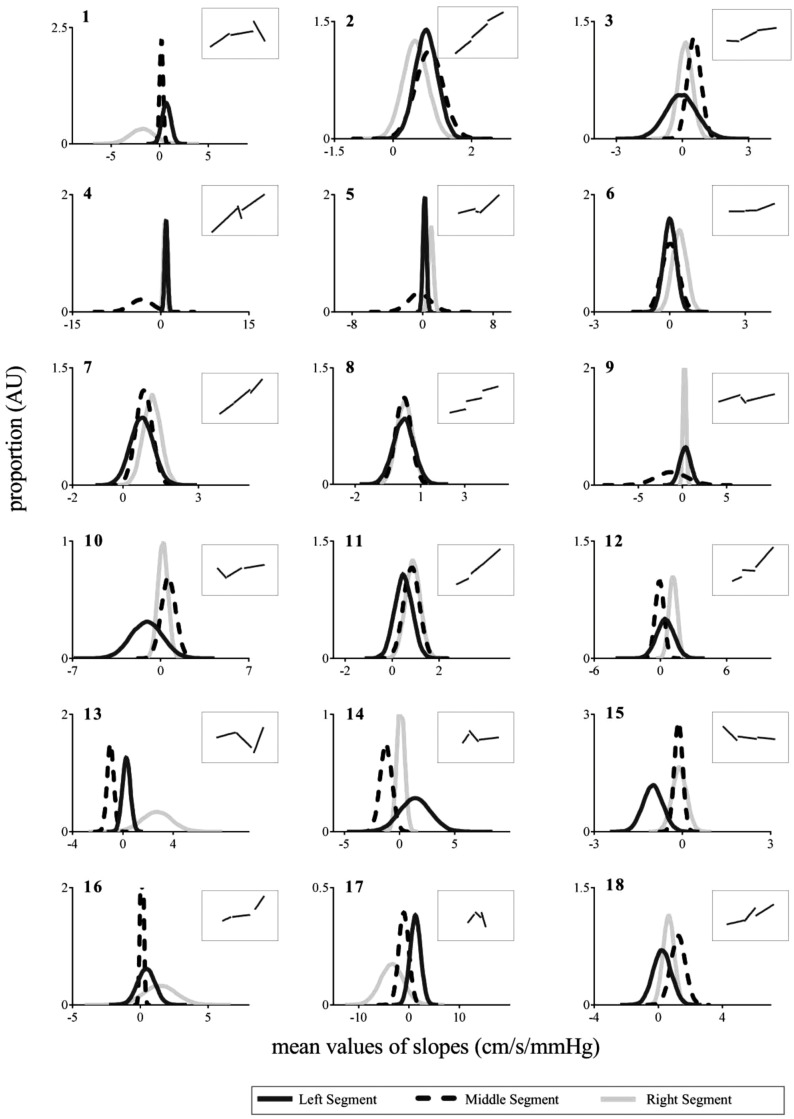
Normal probability distributions corresponding to gradients and their uncertainties of 0.23-Hz resampled data at 0.03Hz OLBNP using unhinged piecewise regression. The corresponding unhinged lines are shown in inset of each subplot. In each subplot the top-left digit(s), ***italic-bold***, refers to the subject’s identifier. OLBNP, oscillatory lower body negative pressure; AU, arbitrary units.

Since the hinging of piecewise regression lines can impose structures on the underlying BP-MCAv relationship, we also fitted unhinged piecewise regression lines to the BP and MCAv data points. Fig 3c shows an example of unhinged piecewise regression fitted to 0.23-Hz resampled data points, and Fig 6 shows the probability distributions corresponding to the gradients and uncertainties of unhinged piecewise lines during 0.03Hz OLBNP for all subjects. For clarity the corresponding unhinged regression fits for each subject are shown in the figure insets. Assessment of the slopes of individual segments showed that at low BP (i.e., left segment) 14 subjects had positive gradients, 3 subjects had negative gradients and 1 subject had approximately zero gradient. For the middle segment 8 subjects had positive gradients, 7 subjects had negative gradients and 1 subject had a gradient that was approximately zero. At high BP (i.e., right segment) 14 subjects had positive gradients, 2 subjects had negative gradients and 2 subjects had approximately zero gradients. Thus qualitatively only 4 subjects demonstrated middle segment gradients that were lower than both left- and right segments (i.e., indicative of possible central plateau). However, the corresponding P-values of the test statistic (defined in equation A8) for unhinged piecewise regression were greater than 0.05 (except for two subjects for which the middle segment gradient is negative and large; see S2 Table) under 0.03Hz OLBNP. These results indicate that the null hypothesis (i.e., absence of a middle segment plateau) cannot be rejected for unhinged piecewise lines.

Residual serial correlation was associated with a 5.5±0.4% difference in the standard deviations of estimated probability distributions of the unhinged regression (see S1 File for details). This difference did not substantially affect our overall conclusions since the P-values of the test statistic for unhinged lines (after taking into account the factor of residual serial correlation) were all still greater than 0.05 for all subjects under each OLBNP condition.

## Discussion

### Main findings

This study sought to define the characteristic relationships between BP and CBF in healthy humans. We found that PPR and LOWESS explained half of the MCAv variance during OLBNP at 0.03Hz, suggesting that BP is an important determinant of CBF dynamics. However, BP-MCAv fluctuations characterised using PPR, LOWESS, and piecewise regression methods showed marked between-subject variations. Although we did observe BP-MCAv relationships that resembled a dCA curve in a few subjects, such relations were absent in a significant majority of individuals. These findings suggest that dCA does not appear to be a process that can be readily summarised by any archetypal relationship.

### Non-linear characterisation of BP-MCAv fluctuations

Prior studies using linear transfer function analysis have suggested that dCA is a frequency dependent phenomenon that can be modelled as a high pass filter [8, 9]. However, the cross-spectral coherence between BP and MCAv in the VLF range (0.02–0.07 Hz) is typically lower than 0.4, indicating that less than 40% of the VLF MCAv variance can be linearly explained by fluctuations in BP. To resolve the inherent nature of these nonlinear relationships, various non-parametric approaches with differing theoretical advantages have been applied including the Hilbert-Huang transformation [26], dynamic non-linear Volterra-Kernel models [27], and variants of Volterra-Kernel models such as Volterra-Laguerre network (LVN) [28], Laguerre-Expansion technique (LET) [29] and Principal Dynamic Modes (PDMs) [30]. In general these Volterra-Kernel based techniques have demonstrated good predictive capability but their complex formulations do not give rise to physiologically interpretable parameters.

In contrast, more recent studies have examined cerebral BP-MCAv dynamics using PPR [4, 15]. One apparent advantage of PPR is that inherently nonlinear relationships can be approximated using weighted sums of ridge functions that are amenable to graphical interpretation [31]. Implementation of this approach during mild OLBNP across a range of frequencies (0.03–0.07 Hz) have revealed nonlinear ridge functions that qualitatively resemble static cerebral autoregulation curves with pressure passive regions shouldering an intervening plateau [4, 15]. These nonlinear ridge functions have been interpreted as a direct representation of dCA and ascribed high deterministic influence on cerebral pressure-flow relationships [4].

However, we were motivated to verify the external validity of this model dCA curve for several reasons. First, although the dCA curve has been reported to explain >50% of the relationship between BP and MCAv fluctuations consistently between individuals even between different study days [4, 15], flow plateaus are decidedly absent in signal averaged traces of cerebral blood velocity [32]. Second, the notion that dCA manifests restricted flow plateau about some average BP set point (within the VLF range) implies that dCA is only effective against minor BP deviations (± 5 mmHg). Very low frequency BP excursions exceeding these narrow margins are associated with parallel changes in CBF, implying that cerebral haemodynamics beyond the dCA range resembles a time-invariant system. However, such implications contradicts data from direct microscopy experiments showing that active dCA tends to occur when BP deviations exceed a threshold >10–15 mmHg (i.e., when there is a clear stimulus), and that beyond this threshold dCA maintains a relatively wide operational range [16].

Therefore, in this study we sought to clarify the characteristics relationships between BP and CBF using a range of complementary methodologies that are also amenable to graphical interpretation. Our analysis showed that BP-MCAv relationships defined using LOWESS were comparable to PPR. However, contrary to previous studies [4, 15], we found that the underlying relationships varied markedly between individuals with few exhibiting LOWESS, PPR, or piecewise regression curves that resembled the dCA curve.

### Potential explanations and implications

It is important to emphasise that although nonlinear regression methods like LOWESS and PPR enable the evaluation of nonlinear systems, these methods do not of themselves explicate the physiological mechanisms that might give rise to observable relationships. Therefore, our findings do not imply that dCA is necessarily non-existent; they simply suggest that CBF dynamics at the lower frequencies studied (0.03 and 0.05Hz) are not consistently linked to BP according to any single deterministic nonlinear dCA curve. Indeed since we expect autoregulatory responses to weaken associations between BP and MCAv, it remains entirely possible that dCA is the reason why BP-MCAv relations appear so obscure. Alternatively, latent interactions between dCA and other flow-influencing processes may be relevant although to resolve these issues future studies will need to incorporate the full range of factors that are known to modulate CBF such the Windkessel properties of the cerebrovasculature [33], the partial pressure of arterial PCO_2_ [34], and neurovascular coupling [35, 36].

One potential explanation for the lack of concordance with previous studies [4, 15] may relate to the way BP and MCAv signals were processed. Specifically, MCAv fluctuations are known to lead BP changes within the VLF range. Therefore, previous PPR analyses that may have assumed that BP and MCAv are instantaneously synchronised may yield sub-optimal estimates of the underlying system [4]. To explore this possibility we also repeated our analyses after factoring in possible delay-shifts between BP and MCAv. Doing so predictably increased model fits by ~10–15%, but does not tangibly influence our overall conclusions.

It is also important to recognise that the dCA curve has been described under situations where BP and MCAv signals have been passed through narrow-band filters (of 0.01 bandwidth). Whilst such pre-processing recognises the frequency-dependent nature of cerebral pressure-flow dynamics, the application of a narrow-band filter may remove harmonics generated by nonlinearities such that only linear components of the signal remain. This issue is particularly significant for higher OLBNP frequencies (e.g., 0.07 Hz) where a 0.01 Hz bandwidth becomes a small proportion of the augmented frequency, and may be one reason why we did not observe the characteristic nonlinear dCA curve reported in previous studies [4, 15]. However, the type of filter used is unlikely to be the main explanation for our findings since 1) the bandwidth we applied corresponds to those used in previous studies [4, 15] and 2) our use of comb filters that allow fundamental and harmonics frequencies to pass but removes (unrelated) noise at intermediate frequencies did not reveal characteristic dCA curves.

The observations of this study may have important implications. Our data suggests that the absence of a clear ‘dCA curve’ does not necessarily imply the absence of autoregulatory behaviour and an abnormal state of CBF regulation. Because this study was conducted in healthy, normotensive and non-smoking subjects, it seems reasonable to assume that the relationships between BP and MCAv fluctuations observed are indicative of complexities that are inherent in normal cerebrovascular systems. Additionally, contemporary clinical monitoring protocols rely heavily on the assumption that brachial blood pressure measurements consistently inform the state of end-organ perfusion. However, as our results indicate, BP-CBF relationships in the VLF range can vary dramatically between individuals. Therefore, many clinical treatment paradigms that focus narrowly on the monitoring and manipulation of BP (e.g., acute blood pressure lowering therapy in stroke) may fail to achieve accurate and targeted control of brain perfusion.

### Methodological considerations

The findings of this study need to be interpreted in the context of several methodological considerations. First, an important underlying assumption of LOWESS, PPR and piecewise regression is that the data points under analysis are statistically independent. This creates a challenge because time series data often demonstrate serial correlation particularly at higher sampling rates (e.g., 5-Hz). Due to the embedding of long-term correlations in physiological signals (up to 60 sec for BP [37]), it is not possible to fully eliminate serial correlation. However, serial correlation can be greatly suppressed by systematically assessing the BP and MCAv autocorrelation functions and down-sampling the data [38]. Here we found that a down-sampled rate of 0.23-Hz created times series that were substantively devoid of serial correlation but still satisfied the minimum Nyquist rate required to characterise the BP-MCAv dynamics of interest in this study. We furthermore developed a novel piecewise regression method that explicitly accounted for the serial correlation factor. Together these measures help reassure that our principal conclusions were not simply due to the serial correlation. Because our objective was to graphically characterise the nature of dCA without any *a priori* assumptions, we did not explore the use of nonlinear time series models that can compensate for serial correlation, but require pre-specification of certain nonlinear functions.

Second, it needs to be acknowledged that cerebral perfusion pressure was estimated using indirect methods. Theoretically, cerebral perfusion pressure is calculated from the difference between mean arterial BP and the effective downstream pressure of the cerebral circulation. In this investigation BP was estimated at the level of the finger using the Finapres, which is based on the volume clamp technique [39]. This practice is generally considered acceptable because unlike systolic BP, mean BP is not affected by pulse wave amplification [39]. Further, Finapres and intra-arterial recordings generate quantitatively similar estimates of mean BP spectral powers within frequency ranges that are commonly analysed in cerebral haemodynamic research [40].

Third, TCD measures cerebral blood flow velocity rather than volumetric blood flow and it will be an adequate surrogate of the actual blood flow only when the insonated vessel diameter is considered invariant to time and different experimental conditions. Several studies in humans have proposed that the diameter of middle cerebral artery remains constant despite the fluctuations in BP and blood gases for conditions similar to those of this study [41].

Fourth, in keeping with the objectives of the study, Fourier transform based filtering approaches were used to extract BP and MCAv fluctuations at each OLBNP frequency. This approach assumes that BP and MCAv time-series are stationary signals composed of sinusoidals. However, considering that BP and MCAv signals possess nonlinear properties, more sophisticated approaches based on the wavelet transform and empirical mode decomposition can also be used to extract oscillatory components associated with the induced OLBNP frequency [11, 26]. One advantage of these methods is that they permit characterisation of time-varying phase relationships between BP and CBF, which may be a powerful approach for nonlinear dCA characterisation [11, 42, 43]. Unfortunately such phase relationship could not be accurately assessed in our 0.23 Hz resampled time series given the raw signal recordings were relatively short in duration.

Finally, we found that substantial (~ 50%) MCAv variations within the lowest OLBNP frequencies studies were not explained by BP alone with either PPR or LOWESS. The variance explained by these techniques can potentially be increased by decreasing the bandwidth of the window function of LOWESS or increasing the number of non-linear PPR ridge functions. However, this is tantamount to increasing the numbers of free parameters that can potentially overfit the data, and give rise to models that are difficult to interpret. To avoid overfitting the data with the LOWESS method, the optimal bandwidth of the LOWESS window function was selected using the nearest neighbour bandwidth selection criteria (i.e., *k*-nearest neighbours) [44].

## Conclusion

In summary, we conclude that BP is an important determinant of CBF dynamics but the underlying pressure-flow relations do not readily manifest itself as a single characteristic dCA curve. These findings should lead to renewed efforts towards developing robust methods for characterising dCA, and towards delineating its influence on CBF from other flow-modulating processes such as carbon dioxide reactivity and neurovascular coupling.

## Supporting Information

S1 Fig Comparison of percentage variance-explained by different regression techniques. ***(a)*** regressions applied directly to 0.23-Hz resampled band-pass filtered data, ***(b)*** cross-validation performed on 0.23-Hz resampled band-pass filtered data, ***(c)*** regressions applied directly to 5-Hz resampled band-pass filtered data, ***(d)*** cross-validation performed on 5-Hz resampled band-pass filtered data. Band-pass filter of 0.01 Hz bandwidth was used. Leave-one-out cross-validation approach was applied for all comparisons. All comparisons were performed by two-way repeated measures ANOVA with the experimental condition (i.e., OLBNP frequency) and regression approach as independent factors. P < 0.05 vs. linear regression, P < 0.05 vs. PPR for significant differences at different OLBNP frequencies. Linear, Linear regression; PPR, projection pursuit regression; LOWESS, locally weighted scatterplot smoother; freq, frequency; reg, regression technique; freq × reg, frequency and regression technique interaction term.
(EPS)

S2 Fig Comparison of slopes of hinged lines fitted to PPR and LOWESS curves (shown in Fig 4) of 5-Hz resampled band-pass filtered BP-MCAv data during OLBNP at 0.03Hz. ***(a)*** slopes of left segment, ***(b)*** slopes of middle segment, ***(c)*** slopes of right segment. AU, arbitrary units; BP, blood pressure; MCAv, middle cerebral artery blood flow velocity; LOWESS, locally weighted scatterplot smoother; OLBNP, oscillatory lower body negative pressure; PPR, projection pursuit regression. Error bars represent means±SD.
(EPS)

S3 Fig Comparison of hinged lines slopes of three segments fitted to PPR and LOWESS curves (shown in Fig 4) of 5-Hz resampled band-pass filtered BP-MCAv data during OLBNP at 0.03Hz. (a) slopes of PPR curves, (b) slopes of LOWESS curves. AU, arbitrary units; BP, blood pressure; MCAv, middle cerebral artery blood flow velocity; LOWESS, locally weighted scatterplot smoother; OLBNP, oscillatory lower body negative pressure; PPR, projection pursuit regression. Error bars represent means±SD.
(EPS)

S1 File Derivation of piecewise regression and test statistics for hypothesis testing. (DOCX)

S2 File Comparison of PPR and LOWES. (DOCX)

S3 File Analysis of delay-shifted data. (DOCX)

S1 Table Test statistic and their P-values for hinged piecewise regression of PPR curves (shown in Fig 4) for 5-Hz resampled band-pass filtered (0.01 Hz bandwidth) data for 0.03Hz OLBNP. (DOCX)

S2 Table Test statistic and their P-values for unhinged piecewise regression (shown in insets of Fig 6) for 0.23-Hz resampled band-pass filtered (0.01 Hz bandwidth) data for 0.03Hz OLBNP. (DOCX)
