# Supplementary material for: Non-Linear Characterisation of Cerebral Pressure-Flow Dynamics in Humans
Source: PLoS One. 2015 Sep 30;10(9):e0139470. doi: 10.1371/journal.pone.0139470 (PMC4589242; doi:10.1371/journal.pone.0139470)
Supplement: S1 File — (DOCX) [file pone.0139470.s004.docx]

**S1 File**

**Piecewise regression and test statistics for hypothesis testing**

The mean and standard deviation of the normally distributed gradient of least square line, defined in equation (1), can be obtained by [[24](#_ENREF_24),[44](#_ENREF_44)]

|  | $\mu_{\hat{\beta}_{1}}=\frac{\sum_{n=1}^{N} \left( P\left( n \right)-\bar{P} \right)(V\left( n \right)-\bar{V})}{\sum_{n=1}^{N} \left( P\left( n \right)-\bar{P} \right)^{2}}$ | (A1) |
| --- | --- | --- |

and

|  | $\sigma_{\hat{\beta}_{1}}=\frac{\sqrt{\frac{\sum_{n=1}^{N} {\left( 1-r^{2} \right)\left( V\left( n \right)-\bar{V} \right)}^{2}}{N-2}}}{\sqrt{\sum_{n=1}^{N} \left( P\left( n \right)-\bar{P} \right)^{2}}}$ | (A2) |
| --- | --- | --- |

where$r$ is the correlation coefficient given by

|  | $r=\frac{\sum_{n=1}^{N} \left( P\left( n \right)-\bar{P} \right)(V\left( n \right)-\bar{V})}{\sqrt{\sum_{n=1}^{N} \left( P\left( n \right)-\bar{P} \right)^{2}}\sqrt{\sum_{n=1}^{N} \left( V\left( n \right)-\bar{V} \right)^{2}}}$ | (A3) |
| --- | --- | --- |

For serially-correlated data points, the standard deviation of the normally distributed gradient of the least square line, defined in equation (1), can be obtained by

|  | $\sigma_{\hat{\beta}_{1}}=\sqrt{\frac{\sigma^{2}}{\sum_{n=1}^{N} \left( P\left( n \right)-\bar{P} \right)^{2}}+2\left( \frac{\sigma}{\sum_{n=1}^{N} \left( P\left( n \right)-\bar{P} \right)^{2}} \right)^{2}\sum_{n=1}^{N} \sum_{i=1}^{N-n} \rho^{i}P(n)P(n+i)}$ | (A4) |
| --- | --- | --- |

where $\sigma^{2}=var(\epsilon(n))$ and $\epsilon(n)$ is assumed as AR(1) model of the form

|  | $\epsilon\left( n \right)=\rho\epsilon\left( n-1 \right)+e(n)$ | (A5) |
| --- | --- | --- |

where $\rho$ is serial-correlation between $\epsilon\left( n \right)$ and $\epsilon\left( n-1 \right)$ and $e(n)$ is uncorrelated random variable. $N$is the number of data points in each segment and, $\bar{P}$ and $\bar{V}$ are the means of BP and MCAv, respectively. $var(.)$ is the variance and AR(1) is autoregressive model of order 1. The difference between the values of equation (A2) and equation (A4) shows the effect of residual serial correlation on the standard deviations of estimated probability distributions.

The statistical significance of the gradients of least square lines of piecewise regression is examined by making the following hypothesis

|  | Null Hypothesis $H_{0}$ : $\beta_{1,middlesegment}$ - $\beta_{1,leftsegment}>0$  (i.e., no central plateau is evident)  Alternate Hypothesis $H_{1}$ : $\beta_{1,middlesegment}$ - $\beta_{1,leftsegment} \leq0$ | (A6) |
| --- | --- | --- |

The test statistic for hinged piecewise regression is

|  | $\left( \hat{\beta}_{1,middlesegment} - \hat{\beta}_{1,leftsegment} \right)-(\beta_{1,middlesegment} - \beta_{1,leftsegment})$ | (A7) |
| --- | --- | --- |

The P-values of the test statistic of equation (A7) are determined using Monte Carlo simulation based empirical approach by generating multiple observations of the random data with the same statistical features (i.e., mean and standard deviation) as that of the original BP-MCAv data.

The test statistic for unhinged piecewise regression is

|  | $\frac{\left( \hat{\beta}_{1,middlesegment} - \hat{\beta}_{1,leftsegment} \right)-(\beta_{1,middlesegment} - \beta_{1,leftsegment})}{\sqrt{\sigma_{\hat{\beta}_{1,middlesegment}}^{2}+\sigma_{\hat{\beta}_{1,leftsegment}}^{2}}}$ | (A8) |
| --- | --- | --- |

The test statistic of equation (A8) will have a Student’s *t*-distribution with $N-3$ degrees of freedom. *N* is the number of data points in each segment of time series.
