# Supplementary material for: Non-Linear Characterisation of Cerebral Pressure-Flow Dynamics in Humans
Source: PLoS One. 2015 Sep 30;10(9):e0139470. doi: 10.1371/journal.pone.0139470 (PMC4589242; doi:10.1371/journal.pone.0139470)
Supplement: S2 File — (DOCX) [file pone.0139470.s005.docx]

**S2 File**

**Comparison of PPR and LOWESS**

S1 Fig shows a comparison of the goodness-of-fit for linear regression, PPR and LOWESS techniques applied to 0.23-Hz and 5-Hz resampled band-pass filtered BP-MCAv data during OLBNP at 0.03, 0.05 and 0.07Hz. S1(a) Fig shows that the linear regression (P < 0.05) produced the worst fit whereas PPR and LOWESS gave rise to the comparable model fits at each OLBNP frequency. S1(b) Fig is a performance comparison of these regression techniques when cross-validated using leave-one-out approach. As expected, the explanatory power of all regressions decreased during cross-validation, but in relative terms LOWESS was associated with best fits. S1(c,d) Figs show that similar patterns and trends in model fits were obtained with the 5-Hz resampled data. Cross-validation was also performed by leaving-out-one entire cycle of OLBNP within each subject. This approach was associated with greater uncertainties (average difference = 5.2 $\pm$0.85%) but did not alter the performance trends.

S2 Fig shows a comparison of the slopes of hinged lines fitted to PPR and LOWESS curves (shown in Fig 4) of 5-Hz resampled band-pass filtered BP-MCAv data during OLBNP at 0.03Hz. We observed that slope trends were similar across PPR and LOWESS for all segments (P=0.7392 for left segment; P=0.6064 for middle segment; P=0.9546 for right segment). These observations indicate that PPR and LOWESS are qualitatively and quantitavely similar. S3 Fig shows that slopes vary in three segments across all individuals and indicate that a diversity of pressure-flow relations is found.
