# Supplementary material for: Non-Linear Characterisation of Cerebral Pressure-Flow Dynamics in Humans
Source: PLoS One. 2015 Sep 30;10(9):e0139470. doi: 10.1371/journal.pone.0139470 (PMC4589242; doi:10.1371/journal.pone.0139470)
Supplement: S3 File — (DOCX) [file pone.0139470.s006.docx]

**S3 File**

**Analysis of delay-shifted data**

We repeated our analyses for both 5-Hz and 0.23-Hz resampled delay-shifted BP and MCAV time series. We observed similar performance trends of goodness-of-fit for PPR and LOWESS techniques but with an increase of 10-15% additional variance-explained as compared to un-shifted data. Similarly, heterogeneous LOWESS and PPR curves were observed for delay-shifted data without obvious evidence of a central dCA plateau. The analyses also showed that the P-values of the test statistics (defined in equation A7) for hinged regression and (defined in equation A8) for unhinged regression were greater than 0.05 under OLBNP conditions of this study. In summary, the delay-shifting did not show evidence of central flow plateau and did not change the overall study conclusions.
