# Supplementary material for: Non-Linear Characterisation of Cerebral Pressure-Flow Dynamics in Humans
Source: PLoS One. 2015 Sep 30;10(9):e0139470. doi: 10.1371/journal.pone.0139470 (PMC4589242; doi:10.1371/journal.pone.0139470)
Supplement: S1 Table — (DOCX) [file pone.0139470.s007.docx]

**S1 Table:** Test statistic and their P-values for hinged piecewise regression of PPR curves (shown in Fig 4) for 5-Hz resampled band-pass filtered (0.01 Hz bandwidth) data for 0.03Hz OLBNP

| Subject | Test statistic for  Middle - Left | P-value | | | Test statistic for  Middle - Right | P-value | |  |
| --- | --- | --- | --- | --- | --- | --- | --- | --- |
| 1 | -1.5115 | | 0.1238 | -1.1055 | | | 0.1250 | |
| 2 | 0.1773 | | 0.9231 | 0.4277 | | | 0.8421 | |
| 3 | 0.1123 | | 0.9134 | 0.1312 | | | 0.6299 | |
| 4 | -1.6069 | | 0.0651 | -1.4128 | | | 0.1365 | |
| 5 | -4.0192 | | 0.0114 | -4.6762 | | | 0.0125 | |
| 6 | 0.1798 | | 0.8214 | -0.0033 | | | 0.5120 | |
| 7 | 0.5054 | | 0.3532 | 0.0931 | | | 0.2822 | |
| 8 | 0.9212 | | 0.9421 | 0.7595 | | | 0.9372 | |
| 9 | 0.5272 | | 0.9927 | -0.0716 | | | 0.3654 | |
| 10 | 1.3912 | | 0.5861 | 0.4378 | | | 0.5124 | |
| 11 | 0.1566 | | 0.8489 | 0.0650 | | | 0.6474 | |
| 12 | -0.4023 | | 0.1524 | -0.4789 | | | 0.0854 | |
| 13 | -1.0836 | | 0.0764 | -2.0762 | | | 0.0854 | |
| 14 | -1.0908 | | 0.2481 | -0.8582 | | | 0.187 | |
| 15 | 1.1248 | | 0.9814 | 0.1956 | | | 0.9754 | |
| 16 | -0.1851 | | 0.1468 | -1.3167 | | | 0.1364 | |
| 17 | -1.6797 | | 0.0518 | 0.3980 | | | 0.9534 | |
| 18 | 0.3340 | | 0.8478 | 0.4801 | | | 0.9468 | |
