# Supplementary material for: Non-Linear Characterisation of Cerebral Pressure-Flow Dynamics in Humans
Source: PLoS One. 2015 Sep 30;10(9):e0139470. doi: 10.1371/journal.pone.0139470 (PMC4589242; doi:10.1371/journal.pone.0139470)
Supplement: S2 Table — (DOCX) [file pone.0139470.s008.docx]

**S2 Table:** Test statistic and their P-values for unhinged piecewise regression (shown in insets of Fig 6) for 0.23-Hz resampled band-pass filtered (0.01 Hz bandwidth) data for 0.03Hz OLBNP

| Subject | Test statistic for  Middle - Left | P-value | | | Test statistic for  Middle - Right | P-value | |  |
| --- | --- | --- | --- | --- | --- | --- | --- | --- |
| 1 | -1.0745 | | 0.1425 | 1.5821 | | | 0.9417 | |
| 2 | 0.1545 | | 0.5611 | 0.7312 | | | 0.7662 | |
| 3 | 0.7736 | | 0.7790 | 0.9125 | | | 0.8176 | |
| 4 | -2.2563 | | 0.0144 | -2.1241 | | | 0.0189 | |
| 5 | -0.4776 | | 0.3174 | -1.0176 | | | 0.1573 | |
| 6 | 0.0297 | | 0.5118 | -0.8372 | | | 0.2031 | |
| 7 | 0.1509 | | 0.5598 | -0.6755 | | | 0.2509 | |
| 8 | -0.0197 | | 0.4922 | -0.1322 | | | 0.4477 | |
| 9 | -0.8285 | | 0.2089 | -0.8344 | | | 0.2030 | |
| 10 | 1.2560 | | 0.8922 | 0.6352 | | | 0.7357 | |
| 11 | 0.6710 | | 0.7480 | -0.1308 | | | 0.4481 | |
| 12 | -0.5896 | | 0.2784 | -2.2431 | | | 0.0135 | |
| 13 | -1.1826 | | 0.1205 | -2.0707 | | | 0.0210 | |
| 14 | -1.7815 | | 0.0398 | -2.0952 | | | 0.0201 | |
| 15 | 2.3848 | | 0.9902 | -0.0979 | | | 0.4611 | |
| 16 | -0.5023 | | 0.3083 | -1.1023 | | | 0.1366 | |
| 17 | -1.6035 | | 0.0575 | 0.9560 | | | 0.8282 | |
| 18 | 1.3723 | | 0.9128 | 1.0303 | | | 0.8468 | |
